# Future heat waves over the Mediterranean from an Euro-CORDEX regional climate model ensemble

**DOI:** 10.1038/s41598-020-65663-0

**Published:** 2020-05-29

**Authors:** M. O. Molina, E. Sánchez, C. Gutiérrez

**Affiliations:** 1 University of Castilla-La Mancha (UCLM), Instituto de Ciencias Ambientales, Avenida Carlos III s/n, 45071 Toledo, Spain; 2 University of Castilla-La Mancha (UCLM), Faculty of Environmental Sciences and Biochemistry, Avenida Carlos III s/n, 45071 Toledo, Spain

**Keywords:** Projection and prediction, Environmental impact

## Abstract

Heat waves are among the most relevant extreme climatic events due to their effects on society, agriculture and environment. The aim of this work is to improve our understanding of heat waves over the Mediterranean basin during the 21^*st*^ century from an ensemble of Regional Climate Models (RCMs). Focus has been placed on sensitivities to forcing global models, emissions scenarios and the RCM resolution, being the first work based on Euro-CORDEX simulations to fully analyze future heat waves in the Mediterranean. Heat wave features are studied with Warm Spell Duration Index (WSDI, duration) and Heat Wave Magnitude Index daily (HWMId, intensity). Results indicate a large increase by the end of the century in both intensity and length of heat waves from all emissions scenarios, global models, and regional models at any resolution. Exceptional heat waves observed early on the century could then become normal by the end of this period. Forcing global models and emissions scenarios play a major role. Clear added value on spatial distribution and heat wave indices are obtained from global to regional models dynamical downscaling, related to the important coastal or orographic aspects widely present over the Mediterranean.

## Introduction

By the end of current 21^*st*^ century, the last IPCC report^1^ indicates that the mean global temperature is projected to increase more than 2 °C for the more extreme greenhouse gases emissions scenario hypotheses (the so-called Representative Concentration Pathways, RCPs). Focusing on extreme climatic features, present and recent past conditions studies^2^ have already measured important temperature changes. Larger increases than the ones obtained for the mean temperatures are expected due to global warming for future conditions^3–5^. Extreme events and, in particular, heat waves such as the recent ones (2003, 2010, 2015 or 2018) over Europe^6,7^ are likely to be attributed to this warming conditions^8^. Heat waves, due to the societal vulnerability and their effects over human health^9–14^, infrastructures^15,16^, agriculture^6^ or natural ecosystems^1,17^, are considered among the most relevant extreme climatic events. For climate change projections these phenomena can become even more relevant, pointing to a clear increase in their frequency and duration throughout the 21^*st*^ century^1,18–20^.

Heat wave is defined, according to the World Meteorological Organization^21^, as an extreme weather event with marked warming of the air over a large area that usually lasts from few days to few weeks. It has to be clearly above the usual values and thus, high percentiles are needed to characterize such events^22^. In^23^, a heat wave is defined as a period where at least 6 consecutive days exceeds their respective calendar-day 90^*th*^ percentile of daily maximum temperature. In^24^, it is considered 3 consecutive days over the 98^*th*^ percentile of maximum temperature. There is plenty of specific definitions with slight differences for thresholds or number of consecutive days. In^22,25,26^, it is concluded that there is no perfect or complete method to fully characterize heat waves. Each definition allow us to partially describe heat waves through different magnitudes: the number of days (episode length), the number of events (to distinguish between several short and very long ones that cover similar total days), the magnitude (accumulated temperature exceedance) or the hottest day on the event (peak temperature). Any accurate heat wave definition should include both intensity and duration aspects. Some of the most commonly used indices are HWMId (Heat Wave Magnitude Index daily^27^) for intensity and WSDI (Warm Spell Duration Index^25^) for duration.

From a meteorological perspective, a heat wave event is produced when a high pressure system remains in the same place for a prolonged period of time, that make a heat wave event to last by advecting warm dry air to the affected region^22,28,29^. This situation is enhanced over dry soils or low humidity regions due to the extreme temperatures probability amplification^30–37^. High pressure systems are also related to clear-sky conditions and above-average radiative heating. These typical blocking conditions during summer season can be caused by specific conditions^38^ in the previous spring.

The Mediterranean basin is a region where hot conditions and particularly heat waves are common and relevant extreme climatic features during summer^39–41^. Several studies have already analyzed this region for present climate conditions and for future climate projections^6,23,27,32,33,42–47^. Using an ensemble of up-to-date Global Climate Models (GCMs), an increase in heat wave days, number of events and peak intensities for the Mediterranean basin have been projected for future conditions^48^. This region is among the ones with largest projected changes. Over the Mediterranean region, several projects have been focused on the regional climate analysis by downscaling procedures in the last two decades, starting from the pioneering projects PRUDENCE^49,50^ and ENSEMBLES^51^ to TiPES^52^. For a region such as the Mediterranean, and for a complex procedure such as heat waves, Regional Climate Models (RCMs) seems to be an interesting tool to complement the studies performed with GCMs. In recent years, CORDEX initiative^53^ and, specifically, the Euro-CORDEX^54,55^ and MedCORDEX^56^ projects offer a new set of higher resolution simulations that allow us to improve our understanding on how extreme events can be described for present and future conditions. The analysis of^43^ and^57^ (specifically for the center of Europe) have shown the ability of the Euro-CORDEX simulations to represent heat waves for present conditions, as forced by the ERA-Interim reanalysis^58^. These works indicate also a large spread among RCMs. It is important to notice that modelled extreme temperatures can suffer from temperature biases^59^, and even more in the case of the Mediterranean basin^60^. Bias-correction methods have been proposed to help on this issue^61,62^. Nevertheless, future climate projections focused on heat waves description over certain parts of Europe have been analyzed: over France^26^, Portugal^63^, Central Europe^64^ and eastern Mediterranean^65^.

The main objective of this study is to analyze the capability of the largest available ensemble of RCMs to reproduce future heat wave events over the whole Mediterranean basin. Work novelty is related to the focus over the whole Mediterranean basin, with all the available Euro-CORDEX simulations, with both RCP4.5 and RCP8.5 climate change greenhouse gases emissions scenarios and with both 0.11° and 0.44° spatial resolutions (around 50 and 12 km size, respectively). Uncertainties, limitations and robust features are analyzed by comparing these different forcing global models, horizontal resolutions, emissions scenarios and the ensemble of regional climate models, and the computation of two indices to describe heat waves. Specific focus is set to the comparison between GCMs and RCMs with the aim to quantify the added value of regional climate dynamical downscaling methods related to heat wave events. The interest is focused on the climatological description of heat waves, as a needed first step for a later study of the mechanisms that could be responsible of the obtained changes.

## Results

### Mean heat wave indices for the whole Mediterranean basin: 2071–2100

**Figure 1:**
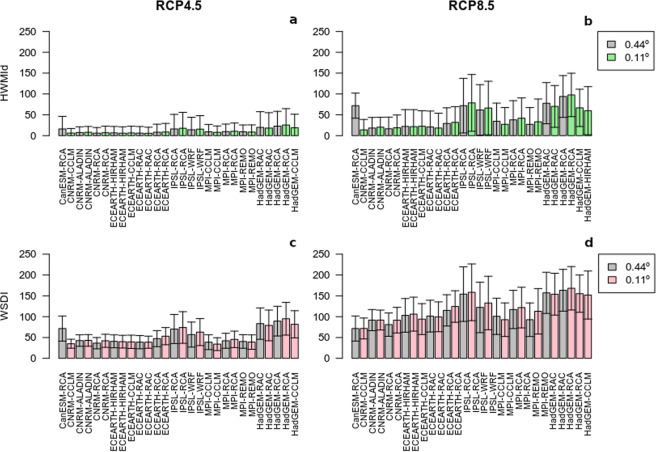
Heat Wave Duration Index daily (HWMId, a and b panels) and Warm Spell Duration Index (WSDI, c and d panels) median and standard deviation (2071–2100) for the whole domain for RCP4.5 (a–c panels) and RCP8.5 (b–d panels) scenarios and 0.11° (coloured bars) and 0.44° (gray bars) resolutions. HWMId is a dimensionless expression of the heat wave maximum magnitude in a year and WSDI is the sum of heat wave days in a year.

Mean annual values of the relative increments of both indices (HWMId, a-b panels, and WSDI, c-d panels) averaged over the whole Mediterranean basin for the 2071–2100 future period, together with the annual standard deviation is shown for each model for both RCPs and horizontal resolutions in Fig. 1. Much larger HWMId and WSDI increments for the RCP8.5 scenario (b-d panels) are obtained when compared with RCP4.5 (a-c panels), which is an expected result^64^ due to its higher radiative forcing. Heat waves are more intense and last longer under the RCP8.5 than in the RCP4.5 scenario, related to the larger increase of mean temperatures. Smaller differences between resolutions (0.44° gray bars and 0.11° green/pink bars) can be observed, which can be mainly related to the averaging result over such a large region (the whole Mediterranean). The global forcing model seems to be of importance. Thus, IPSL and HadGEM exhibit higher heat wave days and magnitude, for both emissions scenarios, as it can be clearly seen when looking at RCA4 regional model, which is forced by all the GCMs. The variability among RCMs show a big spread for both emissions scenarios, in agreement with the results already shown for present climate conditions^43^. Even though, an overall concordance among all RCMs is obtained for the more severe scenario (second column), with more intense and longer heat waves (as in^26^ only focused over France). Both resolutions, 0.11° and 0.44°, show very similar results for both indices, HWMId and WSDI, a result also obtained in^57^. A Mann-Whitney-U test for the averaged values for the whole domain revealed no statistical significance in the difference between median values when compared both resolution results, meanwhile it was significant when compared the two RCPs. This was the result for each model and both indices. With the aim to focus on the main relevant features of heat waves, in the following only higher resolution (0.11°) and emissions scenario (RCP8.5) simulations will be analyzed.

### Mean climatic spatial patterns distribution

**Figure 2:**
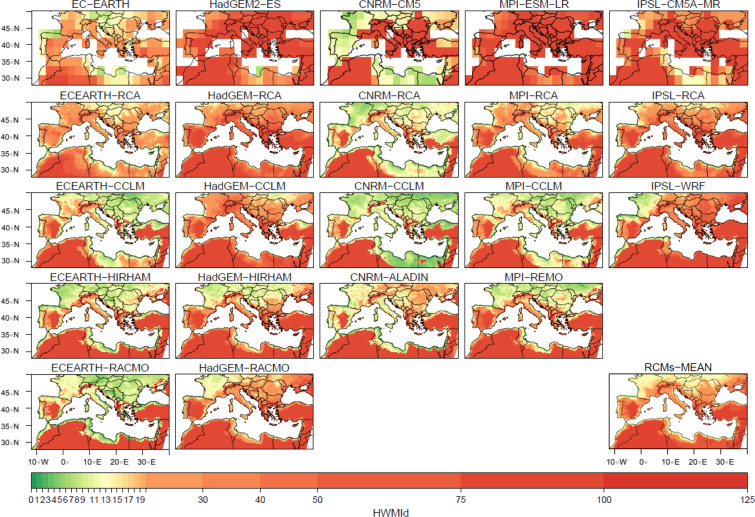
Heat Wave Magnitude Index daily, averaged for the future climate period (2071–2100), for 0.11º resolution and RCP8.5 emission scenario. GCM simulations are presented in the first row in their original resolution. RCM simulations are presented ordered in columns, each one below the GCM that force it. Last figure represents the RCMs ensemble mean. Scale has been truncated to 125, considered as exceptional heat waves.

**Figure 3:**
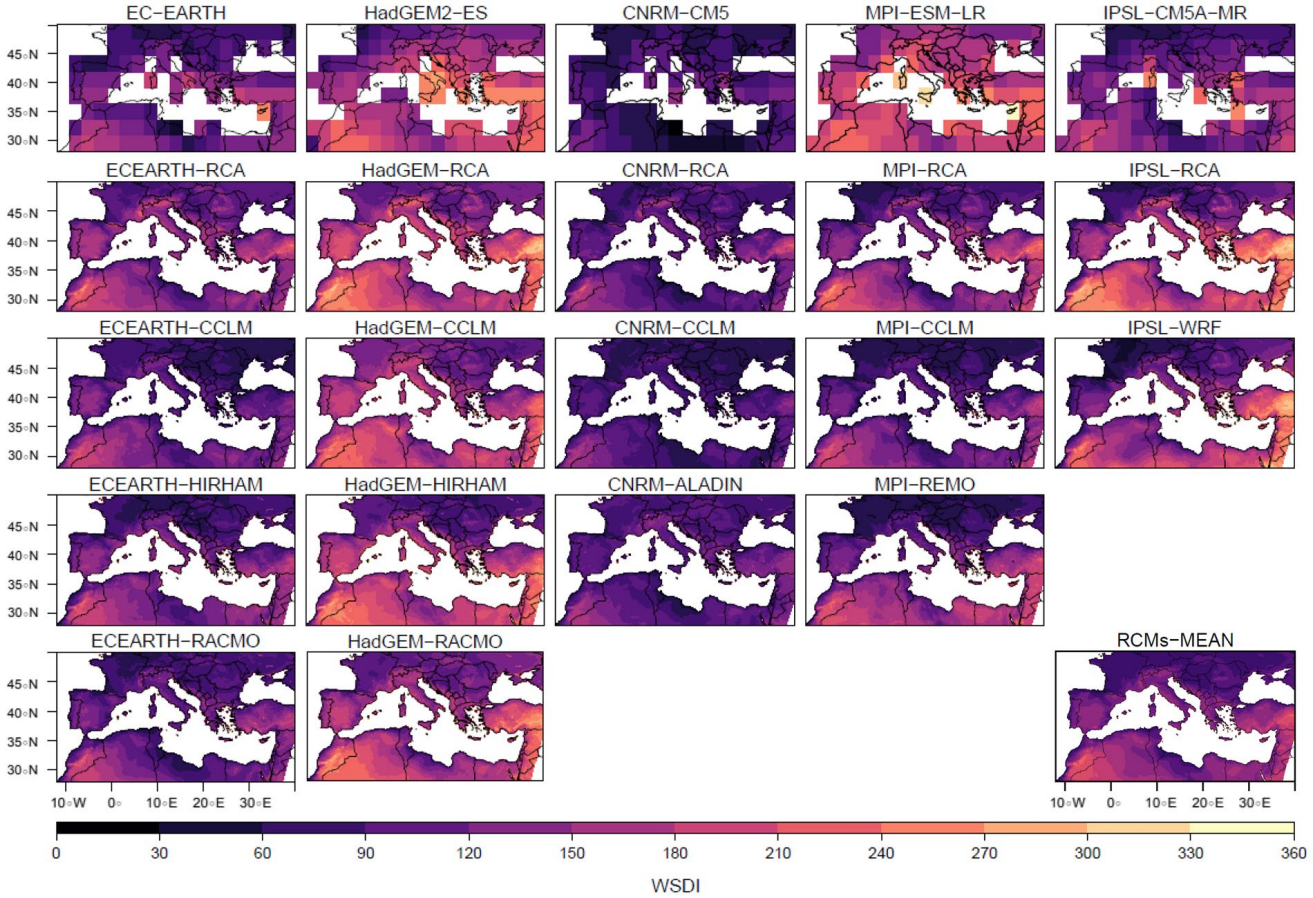
As Fig. 2 for Warm Spell Duration Index (days/year). Scale has been truncated to a value of 360.

The spatial structure of both indices for RCMs at 0.11° resolution and RCP8.5 scenario (averaged for the last 30 years of the 21^*st*^ century) is then presented on Fig. 2 (for HWMId) and Fig. 3 (for WSDI), together with the forcing GCMs. Figures scale has been truncated to 125 for HWMId, as exceptional heat waves^66^, and to 360 for WSDI. The overall patterns of each RCM seem to be first mainly related to their forcing GCM, as column panels (RCMs forced by same GCM) are closer to each other when compared with figures in rows (same RCM forced by different GCMs) on both indices. This is in agreement with the mean values for the whole basin described on previous section with Fig. 1. Among GCMs, CNRM-CM5 is the driving model that shows a less increase for both indices and HadGEM2-ES is the global model with future highest values in the heat wave indices. At the same time, a clear added value from the RCMs when compared with their forcing GCMs can be seen. Many regional features, such as orographic effects over mountainous regions and coastal effects over the Mediterranean basin are obtained^4^. In mountainous regions, heat wave indices exhibit larger increases, meanwhile coastal areas show less heat waves duration and magnitude at the end of century. These results might be due to a better representation of local sea-breeze circulation, and so the transport of cooler and moisture air inland^4^. This potential added value of regional models as a downscaling procedure to improve the representation of extreme events, such as heat waves, have already been pointed by^43,55^. Some studies even indicate that RCMs can help to reduce biases coming from the GCMs including heat waves^67^. When looking at the spatial patterns obtained by the RCMs, related to the low resolution forcing GCMs (first row of Figs. 2 and 3), more detailed patterns and features can be seen, for instance: coastal or orographic effects, which could be of interest for a more precise description of these type of extreme events on regional scales. This has been already pointed by several authors when analyzing heat waves as seen from regional climate model outputs^26,27,30,64^, being these references only focused on RCMs. Furthermore, in^68^ over Africa, it is shown that RCMs were not always able to improve the results of the driving GCMs. Therefore, this work can be seen in these sense as a step forward on RCM/GCM compared analysis, and so, on the studies of potential added value of RCMs. Thus, several regions such as the Iberian Peninsula, exhibit an important north/south and east/west gradients, as the result of the combined influence of the Atlantic ocean and the Mediterranean sea. A relative maximum of the indices is observed in Alps region compared with their surrounding areas^69^ both towards the Italian peninsula and Central Europe. The African coast also exhibits a clear difference between the eastern, central (with a relative minimum over the central African Mediterranean coasts of Libya and Tunisia) and western parts, as already seen in^70^. Sahara region, with the largest values, and mountainous areas over Europe or Turkey with relative maximum values can also be distinguished. All these patterns can be seen as a common signature of a warmer Mediterranean. HWMId and WSDI values would indicate, following previous studies using these indices, that the strongest present climate conditions heat waves could become almost the average values for the end of the century. The 2003 summer heat wave was the second strongest one in Europe since 1950^6^ and reached a peak of HWMId equal to 44.7^27^. Most models agree that this value would be exceeded in west Africa, Turkey and some parts of the Iberian Peninsula and Greece (>100) by the end of the 21^*st*^ century under the RCP8.5 scenario. In^70^, mean values of WSDI between 15–25 days to year in present climate (1981–2010) were obtained for the Mediterranean coastal countries. Our results show mean RCM values between 60–90 heat wave days per year for the northern half of Europe and above 90 days per year for this future period are commonly obtained for most of the coastal areas, no matter which regional model and forcing GCM is used to compute WSDI. This result is in concordance with^54^, where Euro-CORDEX ensemble overview results were presented for the whole continent, including a brief description of heat waves. This agreement can be seen also over subregions such as Central Europe^64^, France^26^ or Africa^70^, despite the fact that a strict comparison is somewhat difficult, as each study uses slightly different indices to describe heat waves. In^64^, it is interesting to notice that in their analysis of heat waves over Central Europe pointed that future heat waves could be correlated with the distribution of maximum temperatures. Here it is shown that both HWMId and WSDI indices reproduce similar features and spatial patterns. Previously, ENSEMBLES regional models simulations, using the former SRES greenhouse gases emissions scenarios, already indicated a similar pattern of large increases^23^.

Despite the described aspects of RCM projections about heat waves increases, some spread among RCMs (when forced with the same GCM) are also obtained, which is consistent with^43^ for present conditions. Some examples of this behavior can be seen in CCLM RCM, that shows less warming in the eastern part of Africa, RACMO22E and WRF331F, that show less warming in France than in the rest of Europe, and ALADIN53, that presents higher warming than the other RCMs driven by the CNRM-CM5 model. Or in the comparison among all the RCA4 RCM combinations, as it is forced by all the GCMs.

### Regional annual timeseries

**Figure 4:**
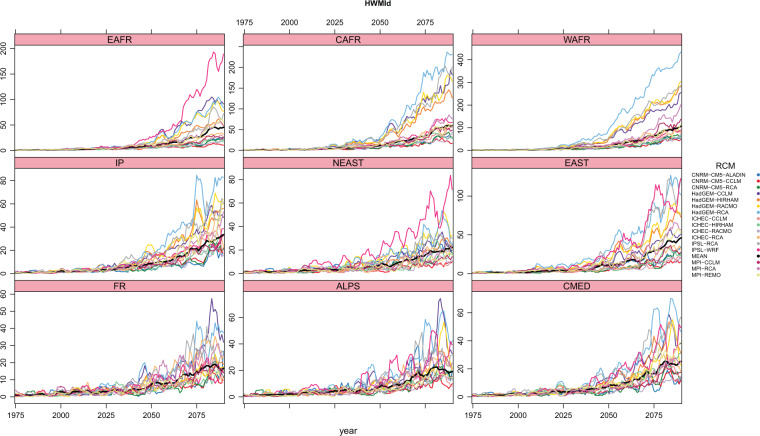
Heat Wave Magnitude Index daily (HWMId) annual values averaged over each of the sub regions described with its corresponding acronym in Supplementary Fig. S1 for each of the RCP8.5–0.11° simulations for the whole 130 year period ranging from 1971 to 2100 in a 5 years running window. The black line refers to the ensemble mean.

**Figure 5:**
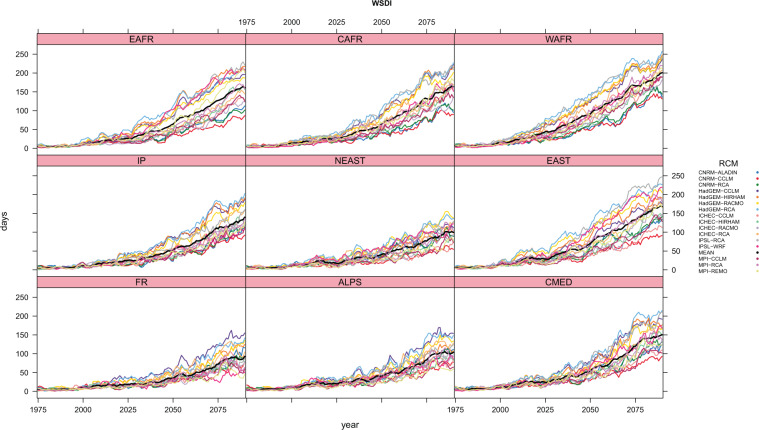
As Fig. 4 for Warm Spell Duration Index (WSDI).

To inspect the time evolution of heat waves throughout the whole 130 period (1971–2100) available from Euro-CORDEX RCM simulations, and with the aim to also study in more detail the subregional features seen on previous section, nine subregions have been defined covering the entire basin, separating the northern, half Mediterranean and African areas, and also the eastern, central and western sides of the basin (Supplementary Fig. S1). These subregions follow roughly what was proposed in the pioneering work about the Mediterranean climate of^42^. In^54^, just the northern side of the Mediterranean basin was considered for a similar analysis. Figure 4 for HWMId and Fig. 5 for WSDI compute the annual time series, in a 5-years running window, for each of the nine regions, for each of the 0.11º-RCP8.5 simulations, together with the ensemble mean of all the RCMs.

HWMId and WSDI values increase during the whole 21^*st*^ century in all regions for all simulations, showing their maximum values by the end of the period. Statistical Mann-Kendall trend analysis with a positive and statistically significant trend (p-value < 0.05, see Supplementary Tables S1 and S2) is obtained for all the regions and simulations. One major result is that the temporal evolution of the indices is different among regions. Each region experience a different evolution both in terms of the magnitude of the change, as well as at the rhythm in which that increase occurs. The increasing trend is complex and non linear with time for each subdomain. Therefore, spatial and temporal heat wave features seem to be quite inhomogeneous. In that sense, the usage of RCMs instead of GCMs could then help on the analysis of the spatial and temporal features more in detail. Regions with a more pronounced increase of both indices are those situated at the east and south of the Mediterranean sea: EAST, WAFR, CAFR and EAFR. All regions exhibit larger heat waves duration increase, from values around 10 days/year during the first part of the period, to values larger than 50. In particular, Western African coast (WAFR) is the region with largest increases (up to 200 days on region average of WSDI, and values higher than 100 of HWMId, in agreement with^66^ findings, where it reaches values of 400 in HagGEM-RCA). This is probably due to the fact that the Saharan desert is part of this area. France area (FR) is the one with smallest increases (about 100 days in WSDI and less than 50 in HWMId), probably related to the fact that includes regions that are far away from the Mediterranean area. These results are coherent with what was seen in Fig. 2 for the 30 years (2071–2100) average. Changes in HWMId values also show a quick increase, similar to what has just been described for WSDI results. It is important to notice also that year-to-year variability is large, being specially clear for the end of the 21^*st*^ century. This result indicates a large interannual variability of heat waves in future climate, as it is the case for current climate. It is remarkable to point that there are also differences among simulations, around what is shown with the curve of the mean value increases, which is logically consistent with the results spread already mentioned before, here in terms of the annual evolution over subregions. Thus, HadGEM-RCA4 and IPSL-WRF tend to show the highest HWMId increases for most of the regions. This is, however, not the case for all the regions. For example, IPSL-WRF clearly presents the highest magnitude values in EAFR and NEAST regions (almost 200 and 80, respectively, at the end of the century), while in WAFR or CAFR just reach medium values (about 60) comparing with the other RCMs. Also, RCM models forced by HadGEM GCM show an increment higher than the others in the three african regions. For WSDI, the same behavior is observed. As an example, HadGEM-CCLM shows the highest trend in annual heat wave days in FR region (with an increment of more than 150 days of heat wave per year to the end of the century), but it shows a smaller trend in the EAST one in comparison with the other RCMs. Regions with lower spread among RCMs are FR and ALPS. On the other side, EAST and WAFR are among the regions with larger dispersion. The comparison between both indices indicate very similar trends with increases in the main characteristics of heat waves, although some small differences have also been detected. WSDI shows a more smoothed trend than HWMId, that begins to increase later and more sharply. Heat waves magnitude (HWMId) begins to increase with higher rates around the year 2071, meanwhile annual heat wave days seem to have already started such increases with respect to the reference period (1971–2000), and so they can be evolving in a slightly different way and rhythm. This is a reasonable result, as they both do not describe exactly the same aspects of heat waves. These results then point to the interest of using more than one index to analyze heat waves. Heat waves are a complex phenomenon, so their complete characterization should be inspected based on the different aspects that define and complement the description of this extreme climatic process.

## Summary and Conclusions

An analysis of heat waves for the Mediterranean basin as seen from Euro-CORDEX ensemble of Regional Climate Model database for future climate conditions in the late 21^*st*^ century has been presented. As described in the introduction, current heat wave events for present climate are becoming more and more important, frequent and relevant. Results shown here are relative increments to the baseline period, so absolute current heat waves are not shown, but only increments related to those events. Therefore further increases obtained from our analysis point to the importance and relevance of these future climate extremes. The results point towards a clear increase by the end of the century, in both intensity and length, of heat waves over the Mediterranean basin in the RCP4.5 and RCP8.5 from any global model and regional model used (including both 0.11º and 0.44º resolutions). In terms of relative importance, greenhouse gases concentration emissions play a major role. The more severe the emissions scenario analyzed, the more intense and lasting heat waves projections will be. As shown by our results, in agreement with previous studies^27,54^, the selection of the GCM that forces the regional models is also relevant. Despite the spread among them, each of the RCMs have a role to describe heat waves, but is less important than the forcing GCM. This spread among RCMs was already indicated by a former study with ERA-Interim reanalysis forcing boundary conditions simulations^43^. Regional climate model resolution (0.11 vs 0.44 degrees size) seems to play a minor role, at least when looking at the overall picture of the whole Mediterranean basin. The analysis presented here is a step forward in a deeper, more complete and detailed analysis compared with the former results of only one index (number of heat waves)^54^, which was made for a sub-sample of the Euro-CORDEX ensemble and analyzed a sub-region of the whole Mediterranean basin. Another relevant feature of the work presented is the comparison between the forcing global models and the regional models, with the aim to look at the potential added value of such dynamical downscaling methodology in terms of both intensity and duration of heat waves. Overall values for large subregions are relatively similar, as it can be expected, but interesting regional features are obtained when going to smaller scales that the RCM are capable to simulate. The Mediterranean basin presents complex orographic characteristics, large land-surface heterogeneities and key and vast coastal processes over the whole basin that are likely to be quite relevant. The obtained results point clearly on that direction, and so to the added-value of using regional climate models related to a more precise description of heat waves over the Mediterranean region.

It has been proved that both WSDI and HWMId heat wave indices are relevant in the analysis and characterization of extreme heat events. The two indexes exhibit consistent results, but also some differences both on the spatial structures and in the time evolution throughout the whole period (1971–2100). For example, annual duration of heat waves (WSDI) seems to start rising earlier than their maximum intensity (HWMId), which would start later, in the middle of the century, and more abruptly. These aspects have not been described before and should be further analyzed in future works. This result point to the interest of using more than one index to better describe heat waves in a more precise way, as duration, magnitude and intensity are all relevant aspects. The subregional analysis indicate that the maximum heat wave increases are obtained in the western African coast (WAFR), probably because part of the Sahara desert is included in the studied domain. France region is the one with less projected heat waves increase at the end of 21^*st*^ century. In summary, the results shown here indicate that by the end of the century the exceptional heat waves measured during the first years of this period in previous works^27,43,71^, could become almost the average values for the end of the century. A better understanding of heat waves over the Mediterranean basin regional environment will be a key aspect on the adaptation strategies needed under the current anthropogenic climate change conditions. Also, the uncertainties and limitations of the current regional climate models on their description are a crucial point to take into account. The next step of the work proposed here would be a detailed analysis of the climatic and atmospheric mechanisms and processes that could be responsible of the obtained changes.

## Methods

### Climate data

The analysis uses the ensemble of RCM simulations available from Euro-CORDEX initiative^54,55^. The Euro-CORDEX simulations consists on multiple dynamical downscaling regional climate models forced by multiple GCMs from the Coupled Model Intercomparison Project Phase 5 (CMIP5)^72^, which is the base of the main climate change projections of the fifth IPCC assessment report^1^. GCMs have a horizontal resolution of about 2 degrees in longitude and latitude for the whole globe. Whilst RCMs from Euro-CORDEX describe the regional climate of Europe at a resolution from 0.44 to 0.11 degrees. Two RCP scenarios are used in this work. They correspond to the stabilization of the radiative forcing by the end of the 21^*st*^ century at certain watts per square meter^73^. The emissions scenarios used here are 4.5 and 8.5 *W*/*m*^2^, being the first related to a moderate emissions scenario and the last one related to the highest forcing scenario. The large amount of Euro-CORDEX available RCM simulations matrix allows to compare different sensitivities and uncertainties due to all the climate modelling chain parts. The simulations cover the 1971–2100 time period, with the European domain fixed by the CORDEX protocol procedures (https://www.cordex.org/domains/).

The combinations of seven RCMs and six GCMs simulations is described in Table 1, where regional models, resolution, emissions scenarios and forcing global models are indicated. A brief description of global climate models can be seen at^54,72^. Daily maximum temperature of the simulations is the used magnitude, as the appropriate variable to compute indices related to temperature extremes^22^. Specific periods are: from 1971 to 2005, defined as historical runs, whereas from 2006 to 2100 are projections forced by the RCPs scenarios. The selected area for this study is the whole Mediterranean basin as shown in Supplementary Material Fig. S1. Models capability to reproduce Euro-CORDEX observed heat waves have already been described in several papers before. In^55^ the main climatic fields are analyzed when forced with ERA-Interim reanalysis and, specifically, for temperature extremes and heat waves on^43^ and^57^. An overview of the main fields for climate change scenarios using the RCPs and the GCM/RCM matrix described above can be seen in^54^. The study is focused directly on climate change results signal, with indices that are related to baseline periods corresponding to present climate conditions.

**Table 1:** List of Euro-CORDEX regional climate model (RCM) simulations using combinations forcing Global Climate Models (GCM), resolution (0.11° or 0.44°) and representative concentration pathways (RCP) greenhouse gases emissions scenarios used for this work.

Acr.	Institute	RCM	GCM	RCP4.5	RCP8.5	0.11°	0.44°
CNRM-ALADIN	CNRM	CNRM-ALADIN53	CNRM-CM5	X	X	X	X
CNRM-CCLM	CLMcom	CCLM4-8-17	CNRM-CM5	X	X	X	
CNRM-RCA	SMHI	RCA4	CNRM-CM5	X	X	X	X
EC-EARTH-CCLM	CLMcom	CCLM4-8-17	EC-EARTH	X	X	X	
EC-EARTH-HIRHAM	DMI	HIRHAM5	EC-EARTH	X	X	X	X
EC-EARTH-RACMO	KNMI	RACMO22E	EC-EARTH	X	X	X	X
EC-EARTH-RCA	SMHI	RCA4	EC-EARTH	X	X	X	X
IPSL-RCA	SMHI	RCA4	IPSL-CM5A-MR	X	X	X	X
IPSL-WRF	IPSL-INERIS	WRF331F	IPSL-CM5A-MR	X	X	X	X
MPI-CCLM	MPI-CSC	CCLM4-8-17	MPI-ESM-LR	X	X	X	X
MPI-RCA	SMHI	RCA4	MPI-ESM-LR	X	X	X	X
MPI-REMO	MPI-CSC	REMO2009	MPI-ESM-LR	X	X	X	X
HadGEM-CCLM	CLMcom	CCLM4-8-17	HadGEM2-ES	X	X	X	
HadGEM-HIRHAM	DMI	HIRHAM5	HadGEM2-ES		X	X	
HadGEM-RACMO	KNMI	RACMO22E	HadGEM2-ES	X	X	X	X
HadGEM-RCA	SMHI	RCA4	HadGEM2-ES	X	X	X	X
CanESM-RCA	SMHI	RCA4	CanESM2	X	X		X

### Heat wave indices

Two indices, Warm Spell Duration Index (WSDI)^25^ for duration and Heat Wave Magnitude Index Daily (HWMId)^27^ for magnitude have been used. WSDI was proposed by an international committee from the World Meteorological Organization, named ETCCDI (Expert Team on Climate Change Detection and Indices^74^), that proposed this magnitude to quantify in days the extension of warm spells in a general sense. It is defined as the number of days per year with at least 6 consecutive days in which the maximum daily temperature is higher than the 90^*th*^ percentile of the maximum daily temperature in a 5 days moving window during the reference period. This period is here defined as 1971–2000. This index considers only the duration of heat waves, therefore, two heat waves with the same duration are considered equally severe, despite having different temperature exceedance from the reference period^70^.

Meanwhile, the Heat Wave Magnitude Index Daily (HWMId)^27,66,71^, is a dimensionless magnitude that was designed to consider both heat wave duration and intensity. It is described as the maximum magnitude of the heat waves in a year. A heat wave is defined from the occurrence during at least 3 consecutive days with daily maximum temperature above the calendar 90^*th*^ percentile centered on a 31 day window related to the reference period (1971–2000), and the maximum magnitude is the sum of the daily magnitudes of each day that compose a heat wave. Both indices show the relative increment for future climate with respect the baseline period of each model employed. More details of the computational procedure of both indices can be found on the references, and on the manual of the free software R packages^75^ used: *extRemes*^76^ for HWMId and *climdex*.*pcic* for WSDI^77^.

Literature is plenty of proposals to characterize heat waves, all of them with advantages and shortcomings. The inspection of how consistent the description from two of the more commonly used indices is also a relevant objective of the work. Multi-annual mean of both indices have been calculated for the whole Mediterranean basin in the far future 30 year period (2071–2100). This is made for each model at both resolutions and both climate change scenarios. Statistical evaluation of the data was carried out with a Mann-Whitney-U test (p < 0.05) in R version 3.6.1 (2019-07-05)^75^ to test the differences in resolution in both scenarios (RCP4.5 and RCP8.5) and the differences between scenarios for both resolutions (0.11° and 0.44°). Annual temporal series are presented for nine subregions, to analyze temporal evolution of the heat waves properties with this spatial detail. The non-parametric test for monotonic trend detection, known as the Mann-Kendall test (from the R package *Kendall*^78^), have been computed to analyze the statistically significance of the trend of those time series, with a 95% confidence level.

## Supplementary information

Supplementary information.
