# Supplementary material for: Future heat waves over the Mediterranean from an Euro-CORDEX regional climate model ensemble
Source: Sci Rep. 2020 May 29;10:8801. doi: 10.1038/s41598-020-65663-0 (PMC7260175; doi:10.1038/s41598-020-65663-0)
Supplement: Supplementary file 1 — Supplementary information. [file 41598_2020_65663_MOESM1_ESM.pdf]

Future heat waves over the Mediterranean from an Euro-CORDEX regional climate model ensemble

M. O. Molina[1,*], E. Sánchez[2] and C. Gutiérrez[1]

[1]University of Castilla-La Mancha (UCLM), Instituto de Ciencias Ambientales, Avenida Carlos III s/n, 45071-Toledo

[2]University of Castilla-La Mancha (UCLM), Faculty of Environmental Sciences and Biochemistry, Avenida Carlos III s/n, 45071-Toledo

[*]MOfelia.Molina@uclm.es

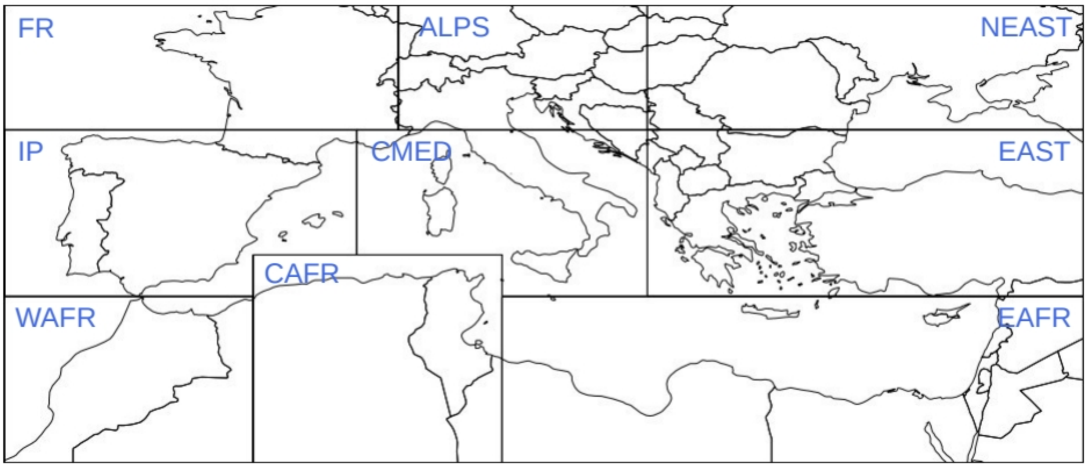

Fig. S1. Description of the nine subregions proposed for details subdomain analysis: FR (France), ALPS (Alps), NEAST (North East Mediterranean), IP (Iberian Peninsula), CMED (Central Mediterranean), EAST (East Mediterranean), WAFR (Western African coast), CAFR (Central African coast) and EAFR (Eastern African coast).

Table S1. Kendall's rank correlation coefficient, tau, of the HWMId annual regional averages for 2071-2100 at 0.11° resolution and RCP8.5 scenario for each single model and ensemble mean (p-value < 0.05).

Model	FR	ALPS	NEAST	PI	CMED	EAST	WAFR	CAFR	EAFR
CNRM-ALADIN	0.60	0.71	0.75	0.68	0.60	0.75	0.85	0.82	0.85
CNRM-CCLM	0.72	0.70	0.58	0.85	0.73	0.73	0.86	0.76	0.86
CNRM-RCA	0.56	0.60	0.69	0.81	0.64	0.71	0.86	0.84	0.85
EC-EARTH-CCLM	0.73	0.68	0.63	0.82	0.80	0.77	0.92	0.87	0.92
EC-EARTH-HIRHAM	0.75	0.70	0.69	0.79	0.72	0.80	0.97	0.84	0.90
EC-EARTH-RACMO	0.75	0.73	0.67	0.79	0.68	0.81	0.88	0.84	0.88
EC-EARTH-RCA	0.76	0.77	0.70	0.86	0.83	0.77	0.91	0.87	0.91
IPSL-RCA	0.78	0.78	0.70	0.86	0.83	0.86	0.94	0.91	0.94
IPSL-WRF	0.70	0.72	0.84	0.85	0.82	0.86	0.91	0.88	0.91
MPI-CCLM	0.66	0.67	0.72	0.85	0.78	0.78	0.90	0.89	0.90
MPI-RCA	0.68	0.78	0.74	0.84	0.81	0.84	0.91	0.88	0.91
MPI-REMO	0.71	0.70	0.71	0.85	0.83	0.84	0.87	0.85	0.87
HadGEM-CCLM	0.83	0.80	0.81	0.91	0.88	0.88	0.94	0.91	0.94
HadGEM-HIRHAM	0.81	0.82	0.81	0.87	0.84	0.86	0.93	0.90	0.93
HadGEM-RACMO	0.72	0.70	0.85	0.87	0.82	0.89	0.94	0.91	0.94
HadGEM-RCA	0.83	0.82	0.86	0.91	0.86	0.90	0.96	0.92	0.96
Mean	0.87	0.90	0.90	0.95	0.92	0.93	0.97	0.96	0.97

Table S2. Kendall's rank correlation coefficient, tau, of the WSDI annual regional averages for 2071-2100 at 0.11º resolution and RCP8.5 scenario for each single model and ensemble mean (p-value < 0.05).

Model	FR	ALPS	NEAST	PI	CMED	EAST	WAFR	CAFR	EAFR
CNRM-ALADIN	0.77	0.83	0.81	0.78	0.81	0.88	0.87	0.87	0.90
CNRM-CCLM	0.76	0.82	0.81	0.82	0.87	0.88	0.91	0.86	0.87
CNRM-RCA	0.76	0.80	0.82	0.82	0.82	0.88	0.90	0.87	0.88
EC-EARTH-CCLM	0.82	0.86	0.78	0.89	0.87	0.87	0.90	0.91	0.92
EC-EARTH-HIRHAM	0.81	0.82	0.81	0.90	0.89	0.88	0.93	0.92	0.91
EC-EARTH-RACMO	0.86	0.86	0.80	0.87	0.85	0.91	0.92	0.91	0.90
EC-EARTH-RCA	0.86	0.88	0.81	0.91	0.90	0.89	0.89	0.88	0.92
IPSL-RCA	0.83	0.88	0.83	0.92	0.93	0.93	0.95	0.93	0.95
IPSL-WRF	0.73	0.82	0.84	0.87	0.88	0.94	0.93	0.92	0.95
MPI-CCLM	0.77	0.78	0.78	0.85	0.85	0.87	0.93	0.91	0.88
MPI-RCA	0.84	0.86	0.81	0.89	0.88	0.90	0.92	0.92	0.90
MPI-REMO	0.81	0.82	0.80	0.87	0.85	0.90	0.91	0.90	0.91
HadGEM-CCLM	0.90	0.90	0.87	0.93	0.91	0.90	0.93	0.93	0.91
HadGEM-HIRHAM	0.88	0.86	0.84	0.91	0.87	0.89	0.94	0.92	0.91
HadGEM-RACMO	0.83	0.85	0.90	0.93	0.87	0.91	0.94	0.93	0.92
HadGEM-RCA	0.90	0.87	0.87	0.93	0.92	0.91	0.93	0.92	0.91
Mean	0.93	0.94	0.93	0.96	0.96	0.95	0.97	0.98	0.97
